# EKG-Diagnostik mit Hilfe künstlicher Intelligenz: aktueller Stand und zukünftige Perspektiven – Teil 2: Aktuelle Studienlage und Ausblick

**DOI:** 10.1007/s00399-022-00855-x

**Published:** 2022-05-12

**Authors:** Wilhelm Haverkamp, Nils Strodthoff, Carsten Israel

**Affiliations:** 1 Abteilung für Kardiologie und Metabolismus. Medizinische Klinik mit Schwerpunkt Kardiologie, Campus Virchow-Klinikum, Charité – Universitätsmedizin Berlin, Augustenburger Platz 1, 13353 Berlin, Deutschland; 2 Berlin Institute of Health Center for Regenerative Therapies (BCRT), Berlin, Deutschland; 3 Department für Versorgungsforschung, Fakultät VI – Medizin und Gesundheitswissenschaften, Universität Oldenburg, Oldenburg, Deutschland; 4 Klinik für Innere Medizin – Kardiologie, Diabetologie und Nephrologie, Evangelisches Klinikum Bethel, Bielefeld, Deutschland

**Keywords:** Elektrokardiographie, Digitale Gesundheitspflege, Maschinelles Lernen, Deep Learning, Künstliche neuronale Netze, Electrocardiography, Digital health, Machine learning, Deep learning, Artificial neuronal networks

## Abstract

Während grundlegende Aspekte der Anwendung von künstlicher Intelligenz (KI) zur Elektrokardiogramm(EKG)-Analyse in Teil 1 dieser Übersicht behandelt wurden, beschäftigt sich die vorliegende Arbeit (Teil 2) mit einer Besprechung von aktuellen Studien zum praktischen Einsatz dieser neuen Technologien und Aspekte ihrer aktuellen und möglichen zukünftigen Anwendung. Die Anzahl der zum Thema KI-basierte EKG-Analyse publizierten Studien steigt seit 2017 rasant an. Dies gilt vor allem für Untersuchungen, die Deep Learning (DL) mit künstlichen neuronalen Netzen (KNN) einsetzen. Inhaltlich geht es nicht nur darum, die Schwächen der klassischen EKG-Diagnostik mit Hilfe von KI zu überwinden und die diagnostische Güte des Verfahrens zu verbessern, sondern auch die Funktionalität des EKGs zu erweitern. Angestrebt wird die Erkennung spezieller kardiologischer und nichtkardiologischer Krankheitsbilder sowie die Vorhersage zukünftiger Krankheitszustände, z. B. die zukünftige Entwicklung einer linksventrikulären Dysfunktion oder das zukünftige Auftreten von Vorhofflimmern. Möglich wird dies, indem KI mittels DL in riesigen EKG-Datensätzen subklinische Muster findet und für die Algorithmen-Entwicklung nutzt. Die KI-unterstützte EKG-Analyse wird somit zu einem Screening-Instrument und geht weit darüber hinaus, nur besser als ein Kardiologe zu sein. Die erzielten Fortschritte sind bemerkenswert und sorgen in Fachwelt und Öffentlichkeit für Aufmerksamkeit und Euphorie. Bei den meisten Studien handelt es sich allerdings um Proof-of-Concept-Studien. Häufig werden private (institutionseigene) Daten verwendet, deren Qualität unklar ist. Bislang ist nur selten eine klinische Validierung der entwickelten Algorithmen in anderen Kollektiven und Szenarien erfolgt. Besonders problematisch ist, dass der Weg, wie KI eine Lösung findet, bislang meistens verborgen bleibt (Blackbox-Charakter). Damit steckt die KI-basierte Elektrokardiographie noch in den Kinderschuhen. Unbestritten ist aber schon absehbar, dass das EKG als einfach anzuwendendes und beliebig oft wiederholbares diagnostisches Verfahren auch in Zukunft nicht nur weiterhin unverzichtbar sein wird, sondern durch KI an klinischer Bedeutung gewinnen wird.

## Abstract (en)

While fundamental aspects of the application of artificial intelligence (AI) to electrocardiogram (ECG) analysis were discussed in part 1 of this review, the present work (part 2) provides a review of recent studies on the practical application of this new technology. The number of published articles on the topic of AI-based ECG analysis has been increasing rapidly since 2017. This is especially true for studies that use deep learning (DL) with artificial neural networks. The aim is not only to overcome the weaknesses of classical ECG diagnostics, but also to extend the functionality of the ECG. This involves the detection of cardiological and noncardiological diseases and the prediction for clinical events, e.g., the future development of left ventricular dysfunction and future clinical manifestation of atrial fibrillation. This is made possible by AI using DL to find subclinical patterns in giant ECG datasets and using them for algorithm development. AI-assisted ECG analysis is becoming a screening tool; it goes far beyond just being “better” than a cardiologist. The progress that has been made is remarkable and is generating much attention and also euphoria among experts and the public. However, most studies are proof-of-concept studies. Often, private (institution-owned) data are used, the quality of which is unclear. To date, clinical validation of the developed algorithms in other collectives and scenarios has been rare. Particularly problematic is that the way AI finds a solution so far mostly remains hidden from humans (black-box character of AI). Overall, AI-based electrocardiography is still in its infancy. However, it is already foreseeable that the ECG, as a diagnostic procedure that is easy to use and can be repeated as often as desired, will not only continue to be indispensable in the future, but will also gain in clinical importance.

Der Trend zum Einsatz von Verfahren der künstlichen Intelligenz (KI) mit ihren Teilbereichen Maschine Learning (ML) und Deep Learning (DL) ist weltweit ungebrochen. Insbesondere letzteres Verfahren kommt auch in der Medizin immer häufiger zum Einsatz [12, 16]. Ein Anwendungsbereich, der derzeit besonders viel Interesse erzeugt, ist die KI-basierte Elektrokardiogramm(EKG)-Analyse. Mit diesem Thema beschäftigt sich die vorliegende zweiteilige Übersicht. In Teil 1 dieser Übersicht wurden die Grundlagen der Anwendung von ML- und DL-Algorithmen besprochen. Der vorliegende Teil 2 widmet sich dem aktuellen Stand der KI-basierten EKG-Analyse, aktuellen Studien zum praktischen Einsatz dieser neuen Technologien und möglichen zukünftigen Perspektiven der Anwendung.

Wie groß das Interesse an der KI-basierten EKG-Analyse ist, spiegelt die Anzahl der in den letzten Jahren zu diesem Thema in PubMed publizierten wissenschaftlichen Veröffentlichungen wider. Seit 2017 steigt sie exponentiell (Abb. 1), vor allem Untersuchungen, die Deep Learning (DL) mit künstlichen neuronalen Netzen (KNN) einsetzen. Inhaltlich geht es in den publizierten Studien nicht nur darum, mit Hilfe von KI die Schwächen der klassischen EKG-Diagnostik zu überwinden und die diagnostische Güte des Verfahrens zu verbessern, sondern auch um den Einsatz des EKG als Verfahren zur Erkennung spezieller kardiologischer und nichtkardiologischer Krankheitsbilder und als Prädiktor für klinische Ereignisse, z. B. der zukünftigen Entwicklung einer linksventrikulären Dysfunktion [3] und von Vorhofflimmern [4]. Die beiden letzteren Modalitäten sind gänzlich neu. Sie machen den Einsatz von KI basierend auf DL erforderlich. Beispiele für aktuelle Studien, die sich mit diesen neuen Themenbereichen beschäftigen, sind in Tab. 1 zusammengestellt.

**Figure 1:**
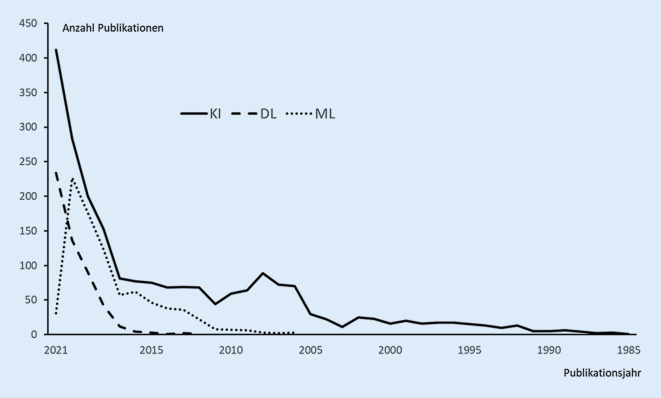

**Table 1:** 

Autor/Jahr	Fragestellung	EKG-Daten	Diagnostische Güte
*Optimierung der traditionellen EKG-Diagnostik*			
Ribeiro et al. 2020 [20]	Automatisierte 12-Kanal-EKG-Analyse (6 Kategorien)	2.1837.12-Kanal-Standard-EKGs von 1.676.384 Patienten, alle EKGs annotiert durch 2 Kardiologen	Sensitivität mit 77–100 % (je nach Kategorie) besser als die von Kardiologen, Notärzten und Medizinstudenten
Strodthoff et al. 2020 [27]	DL-basierte 12-Kanal-EKG-Analyse (unterschiedliche Modelle)	PTB-XL Datensatz: 21.837 EKGs von 18.885 Patienten, annotiert durch Kardiologen	AUC: 0,89–0,96 in Abhängigkeit vom verwendeten KI-Modell
Zhang et al. 2020 [30]	Automatisierte 12-Kanal-EKG-Analyse (18 diagnostische Kategorien)	Trainingsdatensatz: 259.789 EKGs Testdatensatz: 18.018 EKGs	Diagnostische Genauigkeit über alle Kategorien: ca. 95 %; diagnostische Genauigkeit bei der Erkennung eines normalen EKG: 85,49 %; diagnostische Genauigkeit für Vorhofflimmern: 98,27 %
*Identifikation spezieller Krankheitsbilder und extrakardialer Faktoren, die das EKG beeinflussen*			
Ko et al. 2020 [13]	Diagnostik der hypertrophen obstruktiven Kardiomyopathie	Patienten mit mind. einem EKG und einer hypertrophen obstruktiven Kardiomyopathie (diagnostiziert anhand von Standardkriterien) Trainingsdatensatz: 2142 Patienten mit hypertropher obstruktiver Kardiomyopathie und 44.759 Kontrollen	AUC bei Anwendung des Modells auf den Testdatensatz: 0,96; Sensitivität: 87 %, Spezifität: 90 %
Tison et al. 2019 [28]	Diagnostik von kardialen Erkrankungen (pulmonale Hypertonie, hypertrophe Kardiomyopathie, kardiale Amyloidose, Mitralklappenprolaps)	EKGs und Echokardiograme verfügbar EKG-Segmentierung Trainingsdatensatz: 36.186 EKGs von 12.648 Patienten	AUC: 0,88–0,86; im Vergleich mit Maschinenlernen und Kardiologen Bessere Performance; pulmonale Hypertension: AUC: 0,94; hypertrophe Kardiomyopathie: 0,86; Mitralklappenprolaps: 0,77
Galloway et al. 2019 [11]	Erkennung einer Serum-Hyperkaliämie (Serumkalium ≥ 5,5 mmol/l)	EKGs und Serum-Kaliumwerte verfügbar Trainingsdatensatz: 576.581 EKGs von 449.380 Patienten	Validierungsdatensatz: AUC: 0,873 und 0,883
Attia et al. 2019D [6]	Vorhersage von Alter und Geschlecht	Pro Patient 1 EKG Trainingsdatensatz: 399.750 EKGs Validierungsdatensatz: 99.977 EKGs Testdatensatz: 275.056 EKGs	Testdatensatz: mittlerer Fehler bei der Altersschätzung: 6,9 ± 5,6 Jahre; Vorhersage eines Alters ≥ 40 Jahre: AUC: 0,94; Sensitivität: 87,8 %; Spezifität: 86,8 %; Genauigkeit: 87 %; AUC bei der Geschlechtsbestimmung: 0,97
*Vorhersage kardiologischer Krankheitsbilder (Screening)*			
Attia et al. 2019A [3]	Vorhersage einer zukünftig sich entwickelnden linksventrikulären Dysfunktion anhand des EKG	EKGs und Echokardiogram verfügbar Trainingsdatensatz: 35.970 Patienten Validierungsdatensatz: 8989 Patienten Testdatensatz: 52.870 Patienten	Testdatensatz: AUC: 0.93; Sensitivität: 86,3 %; Spezifität: 85,7 %; Genauigkeit: 85,7 %
Attia et al. 2019B [4]	Vorhersage von paroxysmalem Vorhofflimmern bei Sinusrhythmus	Trainingsdatensatz: 454.789 EKGs (126.526 Patienten) Validierungsdatensatz: 64.340 EKGs (18.226 Pateinten) Testdatensatz: 130.802 EKGs (36.280 Patienten)	Testdatensatz: bei Verwendung eines EKGs AUC 0,87; Sensitivität: 79 %; Spezifität 79,7 %; Genauigkeit: 79 %
Baer et al. 2021 [8]	Vorhersage von paroxysmalem Vorhofflimmern bei Sinusrhythmus (retrospektive Analyse)	2412 EKGs, davon 1355 EKGs von Patienten mit paroxysmalem Vorhofflimmern Trainingsdatensatz: 1689 EKGs	Interne und externe Validierungsdatensätze (241 bzw. 1291 EKGs): AUC: 0,79 bzw. 0.75; Sensitivität: 82 % bzw. 77 %; Spezifität: 78 % und 72 %

Bezüglich der Bewertung der Güte der Studien sei auf Teil 1 dieser Übersicht verwiesen

*AUC* Fläche unter der Grenzwertoptimierungskurve

## Automatische Klassifizierung von EKG-Befunden mittels traditionellem ML

Die ersten Arbeiten zur EKG-Analyse mittels traditionellem ML wurden bereits in den 1980ern veröffentlicht. Vor Kurzem sind mehrere ausführliche Übersichten erschienen, die den aktuellen Stand zusammenfassen [17, 21]. Im Vordergrund stehen Studien, die sich mit der automatisierten EKG-Interpretation beschäftigen. Hierbei wird bevorzugt überwachtes Lernen eingesetzt, d. h. die EKGs sind bereits mit Diagnosen versehen. Als Goldstandard bei der Diagnosestellung wird die Auswertung durch Kardiologen angesehen. Dass es auch unter Kardiologen große Unterschiede in der Expertise bei der EKG-Auswertung gibt, bleibt dabei meistens unberücksichtigt. Eine häufig für diese Analysen genutzte Datenbasis stellen die in Teil 1 dieser Übersicht bereit aufgelisteten frei im Internet zugänglichen EKG-Datenbanken dar. Die Güte der ML-basierten Klassifizierung, gemessen anhand der AUC (d. h. der Fläche unter der ROC-Kurve, vgl. Teil 1) übersteigt oft 90 % und schlägt nahezu immer den auswertenden Arzt. Insgesamt ergeben sich aber doch Schwachpunkte, die dazu geführt haben, dass eine klinische Implementierung der auf traditionellen ML-basierten Algorithmen bislang so gut wie ausgeblieben ist. Problematisch sind u. a. der große Aufwand bei der Signalaufarbeitung, lange Berechnungszeiten und Probleme bei der Generalisierung der Modelle. Letzteres ist nicht selten durch Overfitting bedingt, d. h. einer Überanpassung des Modells auf die Trainingsdaten und ein schlechteres Abschneiden hinsichtlich der Vorhersage bei Testdaten [19]. Die meisten Arbeiten stammen aus dem Bereich der Ingenieurswissenschaften und scheinen hier ein gewisses Eigenleben zu führen – die Modelle werden zwar mit viel Mühe erstellt, eine Validierung – als Voraussetzung für den praktischen Einsatz – findet aber so gut wie nicht statt. Das Interesse der EKG-Geräte-Industrie an solchen ML-basierten Algorithmen ist bislang eher gering. Die Hersteller von EKG-Geräten halten an der seit vielen Jahren etablierten klassischen automatisierten EKG-Auswertung fest, obwohl sie mit einer vollkommen unakzeptablen Fehlerrate (20–30 % bei der Diagnostik von Rhythmusstörungen) behaftet ist [23]. Hier mag eine Rolle spielen, dass die Gewinnmargen bei klassischen 12-Kanal-EKG-Geräten so gering geworden sind, dass sich ein Überarbeiten der Gerätetechnik mit Implementierung neuer KI-basierter Auswertealgorithmen, die den Kauf neuer Geräte begründen würde, nicht zu lohnen scheint [18]. Es ist nicht unwahrscheinlich, dass sich dies in den nächsten Jahren in Zusammenhang mit der unaufhaltsam zunehmenden Anwendung von KI und der Einführung und verstärkten Nutzung neuer EKG-Technologien (Patch-EKG-Rekorder, EKG-fähige Wearables) wieder ändern wird.

## EKG-Analyse mit Hilfe tiefer neuronaler Netze

Der aktuelle Hype um KI ist vor allem durch DL mittels KNN begründet. Hierdurch wird die Verarbeitung umfangreicher und hochdimensionaler Daten ermöglicht (vgl. Teil 1). Nachdem DL seine Stärken im Bereich der automatischen Bilderkennung und der natürlichen Spracherkennung bewiesen hat, wird es neuerdings auch vermehrt im Bereich der EKG-Analyse eingesetzt [7, 25, 26, 29]. Für solche Untersuchungen reichen die bei PhysioNet zur Verfügung gestellten EKG-Datensätze meistens nicht aus. Immer mehr basieren die publizierten Untersuchungen auf den bereits in Teil 1 dieser Arbeit besprochenen privaten Datensätzen großer Institute oder Kliniken. Thematisch stehen zwei Aspekte im Vordergrund. Zum einen geht es, ähnlich wie bei der ML-basierten EKG-Analyse, um die weitere Optimierung der traditionellen EKG-Diagnostik. Zum anderen werden Fragestellungen bearbeitet, die für die Elektrokardiographie neu sind. Es geht um die Identifikation spezieller kardiologischer Krankheitsbilder und die allein EKG-basierte Vorhersage von Krankheitszuständen, z. B. der zukünftigen Entwicklung einer linksventrikulären Dysfunktion oder dem zukünftigen Auftreten von Vorhofflimmern. Tab. 1 führt exemplarisch einige aktuelle Studien zu diesen neuen Themen auf. Die Ergebnisse dieser Untersuchungen zeigen, dass viel mehr Informationen im EKG stecken, als landläufig angenommen wird bzw. im Rahmen der klassischen EKG-Auswertung genutzt werden, und die allein darauf basieren, dass Kriterien erfüllt werden, die sich historisch herausgebildet haben. Die mit solchen Zielereignissen einhergehenden EKG-Veränderungen scheinen zum Teil so subtil und/oder komplex zu sein, dass der klassischerweise das EKG auswertende Arzt gar nicht in der Lage ist, sie zu realisieren bzw. sinnvoll zu nutzen. In diesem Zusammenhang wird auch von subklinischen EKG-Veränderungen gesprochen.

## Weitere, über die Anwendung von ML hinausgehende Optimierung der traditionellen EKG-Diagnostik

Vor dem Hintergrund der zunehmenden Arbeitsbelastung im ärztlichen Bereich und des ebenfalls zunehmenden Bedarfs an elektrokardiographischer Diagnostik, der auch dadurch zustande kommt, dass längere Registrierdauern (mittlerweile oft mehrere Tage) immer mehr zur Regel werden, sind leistungsfähige Algorithmen zur Unterstützung der EKG-Analyse unbedingt wünschenswert. Aktuelle Studien zeigen die Möglichkeiten auf, die sich in Zusammenhang mit einer DL-basierten EKG-Analyse ergeben [20, 27, 30]. Sie ist nicht nur der klassischen computerassistierten EKG-Auswertung, sondern auch der EKG-Analyse mit Hilfe von traditionellem ML überlegen [27]. Leider ist bei den meisten vorgeschlagenen Algorithmen bislang keine klinische Validierung erfolgt. Dies steht ihrer praktischen Anwendung im Weg. Zudem fehlt es an Erklärbarkeit [10]. Diese bezieht sich auf die dem entwickelten Algorithmus zugrunde liegenden Regeln. Erklärbarkeit hilft dem Menschen nicht nur zu verstehen, sondern auch zu vertrauen. Beide Aspekte dürften für die zukünftige Akzeptanz der KI-basierten EKG-Analyse ausgesprochen wichtig sein.

## Identifikation spezieller Krankheitsbilder

Abgesehen von Rhythmusstörungen, deren Definition auf elektrokardiographischen Kriterien basiert, erlaubt die klassische Elektrokardiographie nur selten und sehr eingeschränkt, kardiologische Krankheitsbilder explizit zu diagnostizieren. Aktuell gibt es eine ganze Reihe von Studien, die anstreben, diese Limitation mit Hilfe von DL zu überwinden. Ko und Mitarbeiter [13] beschäftigten sich mit der Frage, inwieweit eine KI-unterstützte EKG-Analyse in der Lage ist, Patienten mit einer hypertrophen Kardiomyopathie (HCM) zu identifizieren. Die HCM ist zwar selten, klinisch aber bedeutsam. Sie ist eine der häufigsten Ursachen für plötzliche Todesfäll im Sport. Unter Verwendung der EKGs von 2500 Patienten mit einer validierten HCM-Diagnose und von mehr als 50.000 alters- und geschlechtsgleichen Kontrollpersonen ohne HCM wurde ein konvolutionales KNN trainiert und validiert, um eine HCM allein auf der Grundlage des EKG zu diagnostizieren [13]. In einer unabhängigen Testkohorte von 612 Patienten mit HCM und 12.788 Kontrollpersonen betrug die AUC des KNN 0,96 (95 % KI 0,95–0,96) mit einer Sensitivität von 87 % und einer Spezifität von 90 %.

Zu einer ähnlich hohen AUC kamen Tison et al. [28]. Unter Verwendung von knapp 35.000 EKGs entwickelten sie ein DL-Modell, das nicht nur Patienten mit einer HCM, sondern auch Patienten mit pulmonaler Hypertonie, einer Amyloidose und einem Mitralklappenprolaps erkennt. Die AUC war bei Patienten mit HCM und pulmonaler Hypertonie mit 0,91 und 0,94 sehr hoch; bei den anderen Erkrankungen war sie mit 0,86 (Amyloidose) und 0,74 (MVP) deutlich niedriger. Die zukünftige praktisch-klinische Bedeutung solcher KI-Modelle dürfte beim Screening liegen.

Eine ähnliche Perspektive ergibt sich für KI-Modelle, die anstreben, Patienten mit einer Aortenklappenstenose [9, 14] und einer Herzinsuffizienz mit erhaltener linksventrikulärer Funktion zu identifizieren [24].

Auch extrakardiale Faktoren beeinflussen das EKG-Bild und können mittels KI anhand des EKG identifiziert werden. Hierzu gehören nicht nur das Geschlecht und das Alter [6], sondern auch Veränderungen der Serum-Kaliumkonzentration. In einer erst kurz zurückliegenden Evaluierung wurde ein Modell auf die Erkennung von Serum-Kaliumwerten von ≥ 5,5 mmol/l trainiert. Es wurden über 500.000 EKGs von fast 450.000 Patienten, bei denen der Serum-Kaliumspiegel zeitgleich bestimmt wurde, verwendet [11]. Die AUC im Validierungsdatensatz lag zwischen 0,873 und 0,883. In einer multizentrischen, externen Validierungskohorte ergab sich eine Sensitivität von 90 % und eine Sensitivität von 89 % [2]. Die Autoren spekulieren, dass der Algorithmus eine klinisch bedeutsame Hyperkaliämie ohne Blutabnahme erkennt und zum Screening auf Hyperkaliämien eingesetzt werden könnte.

## Vorhersage kardialer Funktionsstörungen

Das Management der chronischen Herzinsuffizienz mit reduzierter systolischer Funktion stellt eine der großen Herausforderungen der gegenwärtigen Medizin dar. Es mangelt an Möglichkeiten der Frühdiagnose. Grundsätzlich ist diese zwar echokardiographisch möglich, das Verfahren ist aber personal- und kostenintensiv. Ein einfach einsetzbares Screeningverfahren, das in der Lage ist, Patienten mit einer eingeschränkten linksventrikulären Funktion zu identifizieren, bevor Symptome einer Herzinsuffizienz vorliegen, fehlt. Aktuelle Untersuchungen deuten darauf hin, dass sich dies ändern könnte. Attia und Mitarbeiter [3] konnten kürzlich zeigen, dass es anhand des EKG möglich ist, eine sich entwickelnde linksventrikuläre Dysfunktion mittels EKG vorherzusehen. Das Trainieren des zur elektrokardiographischen Diagnostik einer linksventrikulären Funktionseinschränkung (Ejektionsfraktion ≤ 35 %) verwendete tiefe neuronale Netz erfolgte anhand von rund 45.000 Datensätzen. Zu jedem Patienten bzw. EKG stand eine innerhalb von 14 Tagen durchgeführte Echokardiographie zur Verfügung. Die Testung des Algorithmus erfolgte mithilfe weiterer rund 53.000 Patienten. Die Fläche unter der ROC-Kurve betrug 0,93, die Sensitivität 93 %, die Spezifität 86,3 % und die Genauigkeit 85,7 %. Vor dem Hintergrund, dass der Nachweis einer linksventrikulären Funktionsstörung eher als eine Schwäche und nicht als eine besondere Stärke der traditionellen Elektrokardiographie angesehen wird, sind diese Ergebnisse ausgesprochen erstaunlich. Aktuell beschäftigt sich die Arbeitsgruppe mit der klinischen Validierung des Algorithmus [1, 5]. Zu ähnlichen Ergebnissen wie Attia kamen Kwon und Mitarbeiter, die neben dem EKG auch klinische Parameter in ihre DL-Modelle mit einbezogen [15].

## Vorhersage von Vorhofflimmern

Vorhofflimmern ist die häufigste behandlungsbedürftige Rhythmusstörung. Die Tatsache, dass es initial oft nur paroxysmal auftritt und schon in dieser Phase eine deutliche Erhöhung des Schlaganfallrisikos resultiert, limitiert die Bedeutung eines EKG erheblich. Über die Möglichkeiten eines Screenings auf asymptomatisches Vorhofflimmern wird viel diskutiert. Eindeutige Vorgaben zum Vorgehen gibt es bislang nicht. Dass es EKG-Veränderungen gibt, die ein erhöhtes Risiko für Vorhofflimmern anzeigen (z. B. eine verbreiterte P‑Welle), ist schon lange bekannt. Traditionelle EKG-Algorithmen versagen hier allerdings und das Wissen über diese diagnostische Möglichkeit bleibt meistens das Wissen einzelner. In einer kürzlich publizierten DL-basierenden Analyse zur Identifikation von Patienten mit bis dahin nicht bekanntem Vorhofflimmern aus EKGs mit Sinusrhythmus wurden knapp 500.000 EKGs von 126.526 Patienten eingeschlossen [4]. 8,5 % der Patienten entwickelten innerhalb von 31 Tagen nach diesem EKG Vorhofflimmern. Das entwickelte tiefe neuronale Netz war in der Lage, die Arrhythmie mit einer Sensitivität von 79 %, einer Spezifität von 79,5 % und einer Genauigkeit von 79,4 % vorherzusagen; die Fläche unterhalb der ROC-Kurve betrug 0,87. In einem begleitenden Editorial wurde diese Untersuchung begeistert aufgenommen. Letztendlich muss aber bedacht werden, dass die Sensitivität allenfalls mäßig ist. Bei jedem 5. Patienten wird das Vorhofflimmern nicht erkannt. Es ist wichtig zu realisieren, dass es bei dieser Untersuchung nicht darum ging, Patienten zu identifizieren, die im Langzeitverlauf Vorhofflimmern entwickeln. Das Ziel war vielmehr, Patienten zu erkennen, die bis dahin nicht bekanntes Vorhofflimmern aufweisen.

Mit der Frage, inwieweit es mittels DL gelingt, bei Vorliegen von Sinusrhythmus das paroxysmale Auftreten von VHF zu detektieren, beschäftigten sich auch Baek und Mitarbeiter [8]. Die diagnostische Güte ihres Algorithmus war vergleichbar mit dem von Attia. Bemerkenswert ist, dass die von Baek und Mitarbeitern vorgestellte Analyse auf nur knapp 2500 EKGs beruhte. Damit stellt sich die Frage, ob tatsächlich immer so große Datensätze notwendig sind, wie sie z. B. in den Untersuchungen von Attia und Mitarbeitern [4] verwendet wurden. Die Untersuchungen von Baek und Mitarbeitern [8] sind ein Beispiel dafür, dass DL auch mit kleineren Datensätzen gelingen kann.

## Probleme und Risiken bei der KI-assistierten EKG-Analyse

Es wurde bereits erwähnt, dass vor allem für die Entwicklung von DL umfangreiche Datensätze notwendig sind. Zu bedenken gilt, dass sich auch in solchen großen Datensätzen Fehler und andere Unzulänglichkeiten einschleichen können, die in der Lage sind, die Leistung der generierten Modelle bzw. deren Generalisierung zu beinträchtigen. In diesem Zusammenhang wird von Bias (Verzerrung) gesprochen [22]. Bias kann z. B. dann entstehen, wenn systematisch Fehler bei der Datenakquirierung gemacht werden oder die Datensätze für das untersuchte Patientenkollektiv nicht repräsentativ sind. Aus diesen Gründen sind beim Einsatz von KI immer qualitativ hochwertige Datensätze notwendig, die sorgfältig hinsichtlich ihrer Eignung für KI-Anwendungen geprüft werden müssen.

Unverzichtbar sind eine sorgfältige Validierung und Zertifizierung von KI. Die Modalitäten, nach denen beide erfolgen sollen, sind gerade Gegenstand intensiver Diskussionen. Zu einer Validierung von Algorithmen gehört nicht nur die Sicherstellung der Übertragbarkeit von KI-Lösungen auf andere Patientenkollektive. Auch die Übertragung auf neue IT-Umgebungen muss gewährleistet sein. KI wird auch in der Lage sein müssen, aktualisiert zu werden und dazuzulernen. Wie dies erfolgen soll, ist bislang unklar. Derzeit herrscht Einigkeit darüber, dass es eine hundertprozentige Richtigkeit von KI-Entscheidungen in absehbarer Zeit nicht geben wird. Viele Experten schätzen, dass es sie nie geben wird. Von der automatisierten EKG-Auswertung ist bekannt, dass eine unkritische Übernahme von falschen Befunden zu unnötigen Untersuchungen und Kosten führen kann. Dies dürfte in gleicher Weise für die KI-basierte Elektrokardiographie gelten – auch der KI-basierte EKG-Befund muss ärztlicherseits überprüft werden. Eine Verbesserung der Erklärbarkeit von KI dürfte das Vertrauen in KI wachsen lassen. Hersteller von KI-basierter EKG-Software übernehmen keine Haftung für Fehlentscheidungen ihrer KI. Dies muss dem Anwender bewusst sein.

## Schlussfolgerungen

KI hat mittlerweile auch die Elektrokardiographie erreicht. Die verfügbaren Studien sind insofern spannend, als es nicht nur um eine Optimierung der klassischen Elektrokardiographie geht, sondern ganz neue Wege der elektrokardiographischen Diagnostik beschritten werden. Auch wenn die KI-basierte EKG-Diagnostik derzeit noch in den Kinderschuhen steckt, ist absehbar, dass das EKG als einfach anzuwendendes und kostengünstiges diagnostisches Verfahren mit Hilfe dieser neuen Technologien zukünftig für den Arzt wieder interessanter und wichtiger werden wird. Der Einzug von KI in die medizinische Diagnostik wird wohl in den nächsten Jahren unaufhaltsam voranschreiten, dies gilt auch für die Elektrokardiographie. Ersetzen wird KI den Arzt aber auch in diesem Bereich auf absehbare Zeit nicht.
