# Erratum zu: EKG-Diagnostik mit Hilfe künstlicher Intelligenz: aktueller Stand und zukünftige Perspektiven – Teil 2: Aktuelle Studienlage und Ausblick

**DOI:** 10.1007/s00399-022-00871-x

**Published:** 2022-06-02

**Authors:** Wilhelm Haverkamp, Nils Strodthoff, Carsten Israel

**Affiliations:** 1 Abteilung für Kardiologie und Metabolismus. Medizinische Klinik mit Schwerpunkt Kardiologie, Campus Virchow-Klinikum, Charité – Universitätsmedizin Berlin, Augustenburger Platz 1, 13353 Berlin, Deutschland; 2 Berlin Institute of Health Center for Regenerative Therapies (BCRT), Berlin, Deutschland; 3 Department für Versorgungsforschung, Fakultät VI – Medizin und Gesundheitswissenschaften, Universität Oldenburg, Oldenburg, Deutschland; 4 Klinik für Innere Medizin – Kardiologie, Diabetologie und Nephrologie, Evangelisches Klinikum Bethel, Bielefeld, Deutschland

**Erratum zu:**

**Herzschr Elektrophys 2022**

10.1007/s00399-022-00855-x

In der Originalversion des Beitrags stand der Hinweis zu Teil 2 im Untertitel. Dieser Hinweis wurde nun in den Haupttitel eingefügt.

Der Originalbeitrag wurde korrigiert.

